# A new approach to dealing with the analysis of cutpoints in time to event analyses, designed to correct for the over-inflation both of significance levels and of survival differences

**DOI:** 10.1186/1745-6215-14-S1-P107

**Published:** 2013-11-29

**Authors:** Walter Gregory, Susan Burchill

**Affiliations:** 1 University of Leeds, Leeds, West Yorkshire, UK

## Background

Continuous variables, such as biomarkers, are often analysed by comparing survival or other event times by dividing the data above and below a particular cutpoint. The cutpoints are often defined pragmatically, using medians, quartiles or values chosen from other reported analyses. Choosing the most significant cutpoint leads to an over inflation of significance levels and effect sizes. A more statistically sound approach is to consider transformations of these variables. However, clinicians often wish to use different treatments based on the values of such biomarkers, and therefore need appropriate cutpoints on which to base these choices.

## Aim

To develop more rigourous and statistically sound methods for the choice and analysis of cutpoints, as described.

## Methods

Simulations were developed to evaluate whether the effect of the biomarker on the event time was really a threshold effect, which would be optimally described by a cutpoint. Further simulations were used to adjust the significance level to allow for optimising the cutpoint. A method was developed for then adjusting the resulting survival curves comparing values above and below the cutpoint for the fact that the cutpoint had been optimised.

## Results

Examples will be given from actual clinical data/trials to demonstrate the application of these methods, with associated issues.

## Conclusions

Methods have been developed to deal with the real-life and very common need to choose and analyse cutpoints in clinical trial data, which can potentially avoid studies leading to a plethora of different cutpoint analyses that become very difficult to reconcile and interpret.

